# Piglets can secrete acidic mammalian chitinase from the pre weaning stage

**DOI:** 10.1038/s41598-020-80368-0

**Published:** 2021-01-14

**Authors:** Kiyonori Kawasaki, Tomomi Osafune, Saya Tamehira, Kiminobu Yano

**Affiliations:** 1 Faculty of Agriculture, Kagawa University, Kagawa, 761-0795 Japan; 2 University Farm, Kagawa University, Kagawa, 769-2304 Japan

**Keywords:** Animal physiology, Sustainability, Entomology

## Abstract

Fishmeal substitutes (such as insect-based feeds) in pig diets can promote sustainable pork production. Insect powders contain chitin, a nitrogen-containing indigestible material, and pigs must have the capacity to secrete chitin-degrading enzymes to benefit from these diets. The chitin-degrading enzyme (acidic mammalian chitinase; *AMCase*) and its gene expression have been detected in the stomach tissue of approximately 6-month-old fattening pigs; however, it remains unclear from which stage chitin-degrading enzymes are secreted. In the present study, the stomach tissue of piglets was collected from the suckling stage (14 d old) to 56 d to evaluate chitin-degrading enzymes and associated gene expression. *AMCase* mRNA and protein expression was detected in the stomach tissue of all piglets from days 14 to 56. *AMCase* secretion might increase with the increase in stomach tissue weight as piglets grow. Insect powders can therefore be used in the diets of pre-weaning piglets. The gastric *AMCase* level was approximately 30% that of fattening pigs. The appropriate inclusion of insect meals in the diets of pigs at different growth stages still needs to be determined.

## Introduction

Since the publication of "Edible insects" by the Food and Agriculture Organization in 2013^1^, insects have garnered the attention of researchers as potential feed material globally. Food wastes, which are mostly incinerated or landfilled, can be converted to animal proteins by using them as a feed for insects^2^. In addition, the utilization of insect larvae for recycling resources is consistent with the Sustainable Development Goals, and many firms have been set up worldwide to produce insects as a feed source^3,4^.

Some insects that are being studied for their potential use as animal feeds are the mealworm (*Tenebrio molitor*) and black soldier fly (BSF, *Hermetia illucens*)^5–9^. The mealworms have been produced as feed for pets (e.g., birds and reptiles) for a long time^10^, and therefore, their husbandry and breeding methods have been well established^11^. Similarly, the BSF has attracted considerable attention as a potential animal feed and in waste management during recent years^3,7,8,12–15^, and its larvae can be cultivated using either food waste or livestock feces; however, the reproductive efficiency of adult BSFs is low^16^.

The amino acid contents of insect powders are similar to those of fishmeal. Thus, defatted powders of such insects could be used as alternatives to fishmeal, whose availability is influenced by natural resource status, as a protein source in pig and poultry diets^13^. In addition, the supplementation of pig diets with insect powder has been reported to enhance growth and nutrient digestibility with no detrimental effects on the immune system in piglets^17,18^.

The insect cuticle consists of chitin, proteins, phenolic compounds, and lipids^19^. Chitin is a nitrogen-containing fiber^20^ that can only be degraded by chitinase^21^. Numerous studies have reported the immunomodulatory effects of chitin^22,23^. According to these previous studies, the immunomodulatory effects of chitin implied that chitin is not fully digested and eventually reaches the cecum and colon. The substitution of an easily digestible protein source with insect powder, which is relatively difficult to digest, could decrease the growth rates of piglets that have an immature digestive system; therefore, caution should be exercised when adopting insect powder as a protein source.

Mammals produce two types of chitinase, chitotriosidase (*Chit1*) and acidic mammalian chitinase (*AMCase*)^24^. Tabata et al.^24^ reported *AMCase* expression in the stomach tissue of approximately 6-month–old pigs. This suggests that mature pigs have the ability to secrete chitinase. However, it remains unclear from which growth stage chitinase is secreted. Therefore, in the present study, we investigated whether piglets have the capacity to secrete chitinase, from the suckling to growing stages.

## Results

### Body and stomach tissue weights of piglets

The body weight of the piglets at birth was 1344.4 ± 312.3 g. The piglets weighed > 1 kg at birth, and they were not runts. The body and stomach tissue weights at different ages are listed in Table 1.

**Table 1:** Body and stomach tissue weights of the piglets at different age.

Age	Male (n = 3)		Female (n = 3)		All (n = 6)		ANOVA of body weight			ANOVA of stomach tissue weight		
	Body weight (kg)	Stomach tissue weight (g)	Body weight (kg)	Stomach tissue weight (g)	Body weight (kg)	Stomach tissue weight (g)	Age	Sex	Age × sex	Age	Sex	Age × sex
14d	5.0 ± 0.4	21.1 ± 2.3	4.1 ± 0.3	19.2 ± 1.1	4.5 ± 0.3^a^	20.2 ± 1.2^a^	< 0.01	0.15	0.94	< 0.01	0.15	0.68
21d	6.6 ± 0.8	28.9 ± 3.4	6.0 ± 1.2	31.4 ± 6.8	6.3 ± 0.6^ab^	30.2 ± 3.5^a^						
28d	8.7 ± 0.1	37.2 ± 1.5	6.5 ± 1.2	35.3 ± 1.9	7.6 ± 0.7^abc^	36.3 ± 1.1^abc^						
35d	11.1 ± 1.3	68.3 ± 3.0	10.6 ± 1.0	67.3 ± 2.2	10.8 ± 0.7^bc^	67.8 ± 1.7^bc^						
42d	12.9 ± 1.2	90.0 ± 9.0	12.5 ± 2.3	77.0 ± 4.7	12.7 ± 1.2^c^	83.5 ± 5.4^c^						
49d	21.4 ± 3.8	149.2 ± 28.5	19.9 ± 0.9	137.0 ± 8.7	20.6 ± 1.8^d^	143.1 ± 13.6^d^						
56d	24.9 ± 1.7	185.2 ± 6.3	20.7 ± 4.1	150.4 ± 24.9	22.8 ± 2.2^d^	167.8 ± 13.9^d^						

Data is mean ± SE. Different alphabet characters indicate significant differences at the 0.05 level.

### Chitinase-related mRNA expression in the stomach of piglets

**Figure 1:**
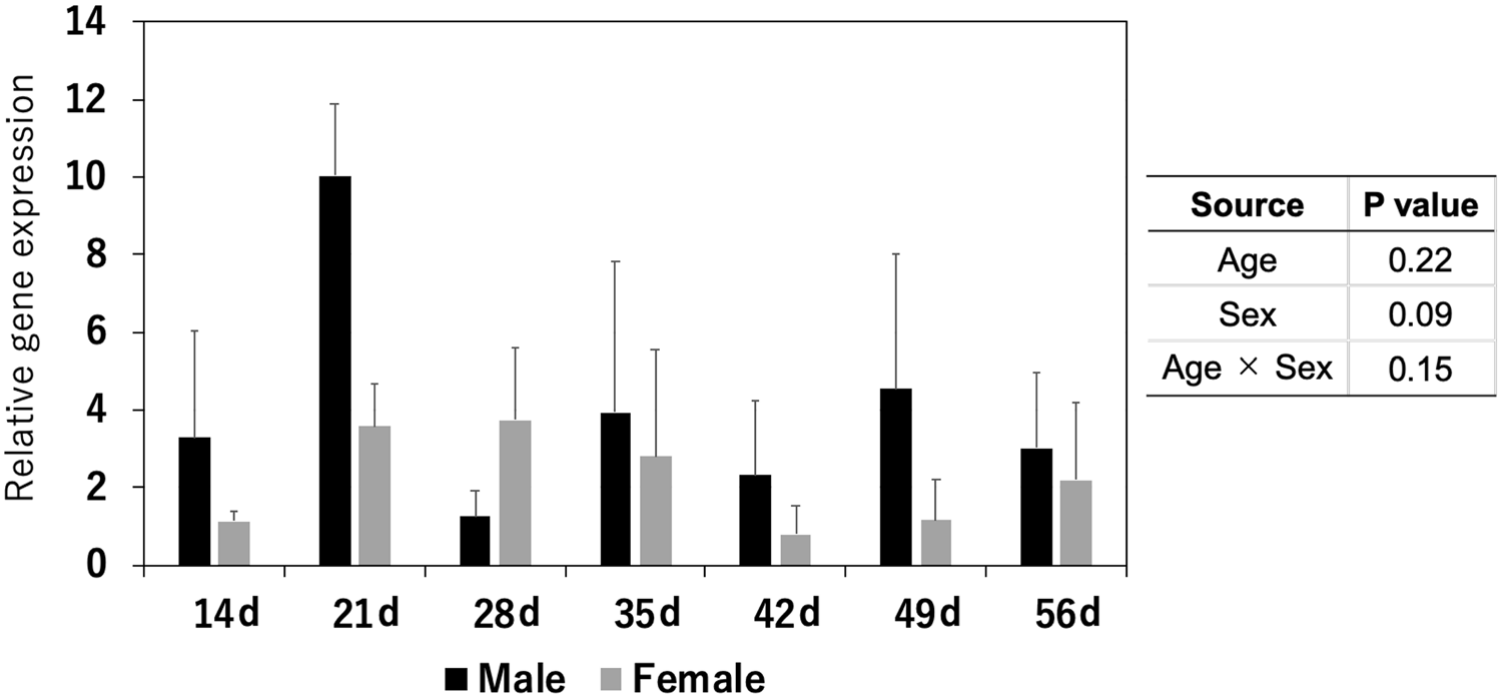
*AMCase* mRNA expression level in the stomach tissue of piglets. Data are presented as mean ± standard error. Values are expressed as relative gene expression.

*AMCase* mRNA was expressed in all piglets. There were no significant differences in the mRNA expression of *AMCase* among piglets of different ages (*P* > 0.05, Fig. 1). Moreover, there was no significant difference in the relative expression of *AMCase* between the sexes of piglets (*P* > 0.05, Fig. 1). *Chit1* mRNA expression was observed in only 4 (two 21-, one 35-, and one 56-d-old) of the 42 piglets, and the threshold cycle (Ct) of *Chit1* was 15–20-fold that of *AMCase*.

### *AMCase* level in the stomach of piglets

**Figure 2:**
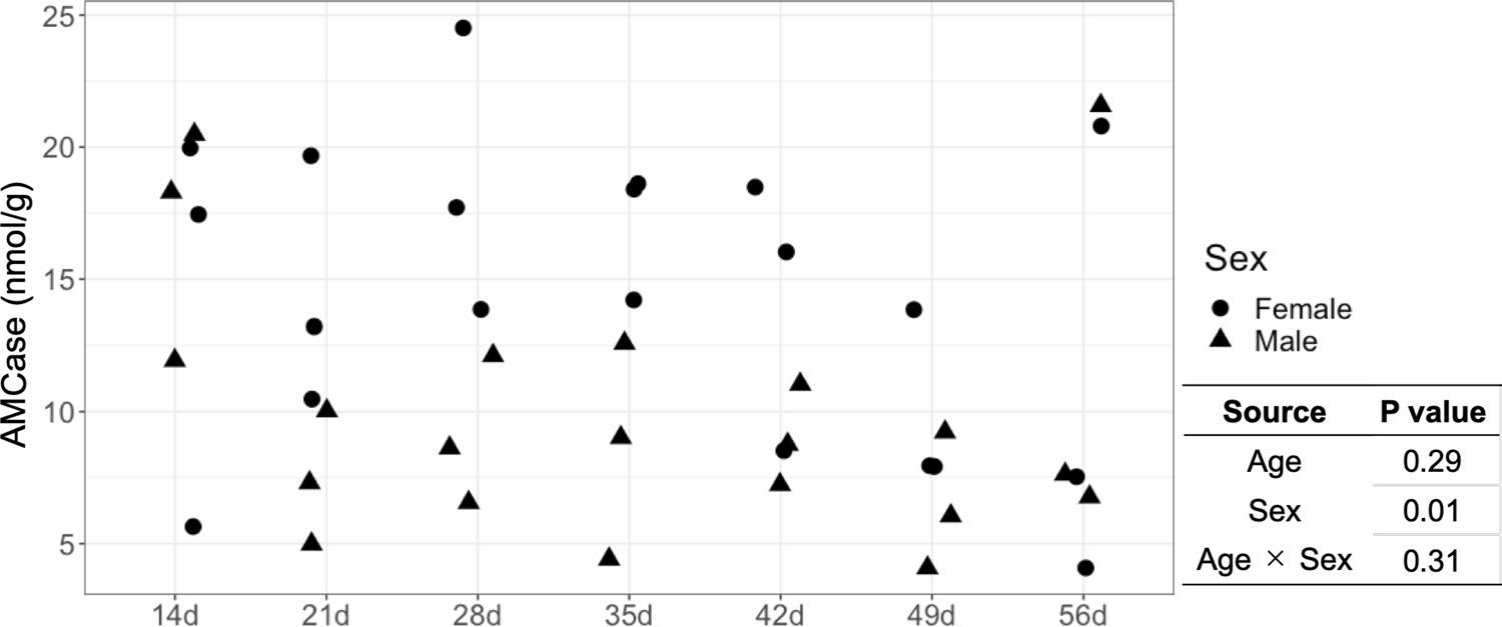
*AMCase* levels per unit stomach tissue weight in piglets.

*AMCase* was secreted in the stomach tissue of all piglets. The *AMCase* levels per unit stomach weight did not differ significantly among piglets of different ages (*P* > 0.05, Fig. 2). However, the analysis of variance (ANOVA) showed a significant difference in *AMCase* level per unit stomach weight between piglet sexes (*P* < 0.05, Fig. 2).

**Figure 3:**
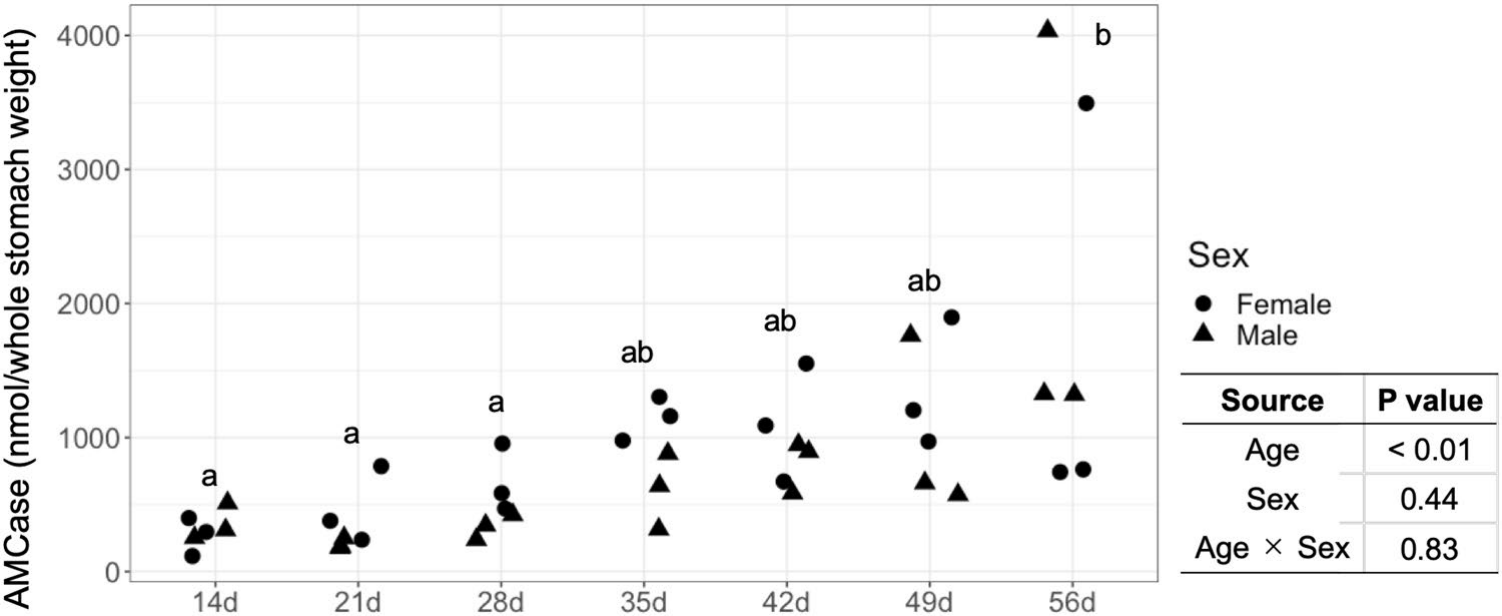
*AMCase* levels in whole stomach weight of piglets. Different lowercase letters indicate a significant difference (Bonferroni post-hoc test, *P* < 0.01).

The *AMCase* level per whole stomach weight tended to increase with an increase in age (Fig. 3). The *AMCase* level in the whole stomach of piglets was significantly higher at 56 d than at 14 d (*P* < 0.05). However, the ANOVA did not reveal significant differences in the *AMCase* level in the whole stomach between piglet sexes (*P* > 0.05, Fig. 3).

## Discussion

In the present study, we aimed to clarify the stage from which chitin-degrading enzymes are secreted. To this end, the stomach tissue of piglets was collected from the suckling (14 d old) to the growing stages (56 d old) to investigate chitin-degrading enzyme and associated gene expression levels. Piglet *AMCase* gene expression did not vary among the growth stages, suggesting that the piglets had the ability to secrete *AMCase* from the suckling stage. Similar to the findings of Tabata et al.^24^, *Chit1* mRNA expression in the stomach was low compared to *AMCase* mRNA expression. In addition, the relative *AMCase* mRNA expression did not significantly change when the piglets began to wean, and the stress of weaning did not seem to influence the gene expression levels considerably. Although the piglets were allowed to consume only maternal milk and water until weaning, the piglets exhibited interest in commercial feed from around 9 d of age when the feed was placed in the rearing enclosures; they have been reported to poke the feed with their noses or eat it^25^. In the wild, piglets probably begin to eat insects and mushrooms that contain chitin. Thus, pigs may have the ability to secrete *AMCase* in the pre-weaning stage.

In the present study, the *AMCase* level did not exhibit an increasing trend with age between the pre-weaning and post-weaning stages; however, it was approximately 28% that in the stomach tissue of fattened pigs^24^. The results suggest that *AMCase* secretion increases with the growth of pigs. As the weight and area of the gastric mucosa increase with pig growth, even if the *AMCase* level per unit area of gastric mucosa is the same, the degraded chitin amount would increase with age. This implies that insect powder concentrations in pig diets can be increased with the growth stage of pigs.

Several studies in which the protein source in piglet diets has been substituted with insect powder have been reported; however, in most of these studies, the protein has been substituted with soybean meal, and a few studies have used animal-based proteins^18,26–28^. In addition, it has been reported that soybean meal can be substituted with 10% insect powder^26^; however, the insect powder used to replace soybean meal was defatted under high pressure and may not contain a considerable amount of chitin. Another study that substituted non-defatted insect powders with animal protein in piglet diets concluded that 2% substitution was possible^29^.

Protein concentrations in pig diets decrease from the pre-weaning stage to the fattening stage, when their diets shift to contain more grains than animal proteins^30^. In addition, based on the composition of the diet, the suckling stage diets contain more animal proteins, but the fattening stage diets contain more grains than animal proteins; therefore, the proportion of animal proteins in the diets of fattening pigs is low^30^. Consequently, animal proteins in the diet of fattening pigs, which are thought to secrete a higher amount of chitin-degrading enzymes than piglets, can be entirely substituted with insect powder. However, as the proportion of animal proteins in the diet during the pre-weaning and early weaning stages are high^30^, it is necessary to consider the chitin level when replacing animal proteins in the diet with insect powder. Otherwise, the chitin nitrogen would be calculated as the amount of nitrogen in the diet, and the amount of piglet digestible nitrogen could be lower than that presumed. Furthermore, inappropriate concentrations of insect powder in diets could inhibit weight gain in piglets.

The piglets exhibited the ability to secrete *AMCase* in the stomach tissue from day 14. Therefore, insect powder could be adopted in the diets of pre-weaning piglets. However, the gastric *AMCase* level in piglets was approximately 28% that in fattening pigs; this necessitates further investigation to determine the appropriate insect powder concentration in the diets at different growth stages.

## Methods

The protocol of this study was approved by the Animal Care and Use Committee of Kagawa University (Permit number: 2019–19,631) and carried out according to the Kagawa University Animal Experimentation Regulation.

### Stomach tissue samples from piglets

Forty-two piglets (Landrace × Large white × Duroc: 21 males and 21 females) delivered by six sows (Landrace × Large white) were used. The sows and piglets were reared in farrowing stalls (1.8 m × 2.0 m) until weaning. Weaning was carried out at 28 d. After weaning, out of the 42 piglets, 24 piglets were reared in piglet cages (1.8 m × 1.8 m) in an open-type pig house with an average temperature of 21.8 °C ± 5.5 °C and average humidity of 70.3% ± 6.5%. The piglets were fed commercial feeds (Nosan, Yokohama, Japan) according to the feeding program of the university farm. The feeding program and the feed compositions are shown in Fig. S1 and Table S1, respectively. At 14 d, six piglets (3 males and 3 females) were euthanized by administering 90.0 mg/kg body weight of pentobarbital via the auricular vein and dissected. This process was repeated weekly for 7 weeks with the remaining piglets. Stomach tissue was collected from the gastric body to the pylorus, and then weighed. The collected samples were separated for gene expression analyses and chitinase measurements. The stomach tissue samples for gene expression analyses were stored at 4 °C overnight in RNAlater stabilizer solution (Thermo Fisher Scientific, Waltham, MA, USA), and then frozen at -80 °C until analysis. The samples for chitinase secretion assays were frozen at -80 °C until analysis.

### Expression of piglet *AMCase*

Total RNA was isolated from piglet stomach tissue using TRIzol Reagent (Thermo Fisher Scientific), according to the manufacturer’s instructions. The RNA was reverse transcribed into cDNA using ReverTra Ace qPCR RT Master Mix with gDNA Remover (TOYOBO, Osaka, Japan), according to the manufacturer’s instructions. The sequences of primers used for real-time PCR were as follows: *AMCase* forward primer 5ʹ-TGACTTCACAGGCACTTTCT-3ʹ, *AMCase* reverse primer 5ʹ-CGGTGCAACTTGTGCTATTC-3ʹ; *Chit1* forward primer 5ʹ-GTCAACTCAGCCATCAGGTT-3ʹ, *Chit1* reverse primer 5ʹ-CAAGGTCAAGGCCATCAAA-3ʹ; and *GAPDH* forward primer 5ʹ-ACCTCCACTACATGGTCTACA-3ʹ, *GAPDH* reverse primer 5ʹ-ATGACAAGCTTCCCGTTCTC-3ʹ^24^. A StepOnePlus Real-Time PCR system (Applied Bioscience, Waltham, MA, USA) was used for real-time PCR, and each reaction mixture consisted of 0.4 µl of 50 × ROX reference dye (TOYOBO, Osaka, Japan), 10 µl of THUNDERBIRD SYBR qPCR Mix (TOYOBO, Osaka, Japan), 2 µl of DNA template, 0.5 µM of forward primer, 0.5 µM of reverse primer, and 5.6 µl of sterile water, with a total volume of 20 µl. Each reaction was performed in triplicate. The PCR cycling conditions were as follows: initial denaturation at 95 °C for 1 min; 40 cycles of denaturation at 95 °C for 15 s, and extension at 60 °C for 1 min.

### Measurement of piglet *AMCase*

Twenty milligrams of gastric mucosa and 1 ml of RIPA Buffer (Nacalai Tesque, Kyoto, Japan) were added into a 1.5 ml tube. Thereafter, the samples were completely homogenized using a bead crusher (µT-12; TAITEC, Saitama, Japan) by shaking at 3,500 rpm for 20 s, and then cooling on ice for 30 s; this process was repeated three times. Subsequently, the samples were centrifuged at 10,000 × *g* for 20 min at 4 °C, and the cells were precipitated. The supernatant was transferred to another 1.5 ml tube without disturbing the pellet.

The *AMCase* level was measured using a fluorometer (Gemini EM; Molecular Devices, San Jose, CA, USA) and the CycLex Acidic Mammalian Chitinase Fluorometric Assay Kit (Medical & Biological Laboratories, Nagoya, Japan), according to the manufacturers’ instructions.

### Statistical analysis

The relative *AMCase* mRNA and protein expression levels were analyzed using a two-way ANOVA and Bonferroni post-hoc test using IBM SPSS Statistics 25 (IBM Corp, Armonk, NY, USA).

## Electronic supplementary material

Below is the link to the electronic supplementary material.

Supplementary Information 1
