# Supplementary material for: Piglets can secrete acidic mammalian chitinase from the pre weaning stage
Source: Sci Rep. 2021 Jan 14;11:1297. doi: 10.1038/s41598-020-80368-0 (PMC7809370; doi:10.1038/s41598-020-80368-0)
Supplement: Supplementary file 1 — Supplementary Information 1 [file 41598_2020_80368_MOESM1_ESM.docx]

**Supplementary Figure and Table**

**Piglets can secrete acidic mammalian chitinase from the pre weaning stage**

Kiyonori Kawasaki^1,*^, Tomomi Osafune^1^, Saya Tamehira^1^, and Kiminobu Yano^2^

^1^ Faculty of Agriculture, Kagawa University, Kagawa, 761-0795, Japan

^2^ University Farm, Kagawa University, Kagawa, 769-2304, Japan

* Corresponding author: kkawasaki@ag.kagawa-u.ac.jp


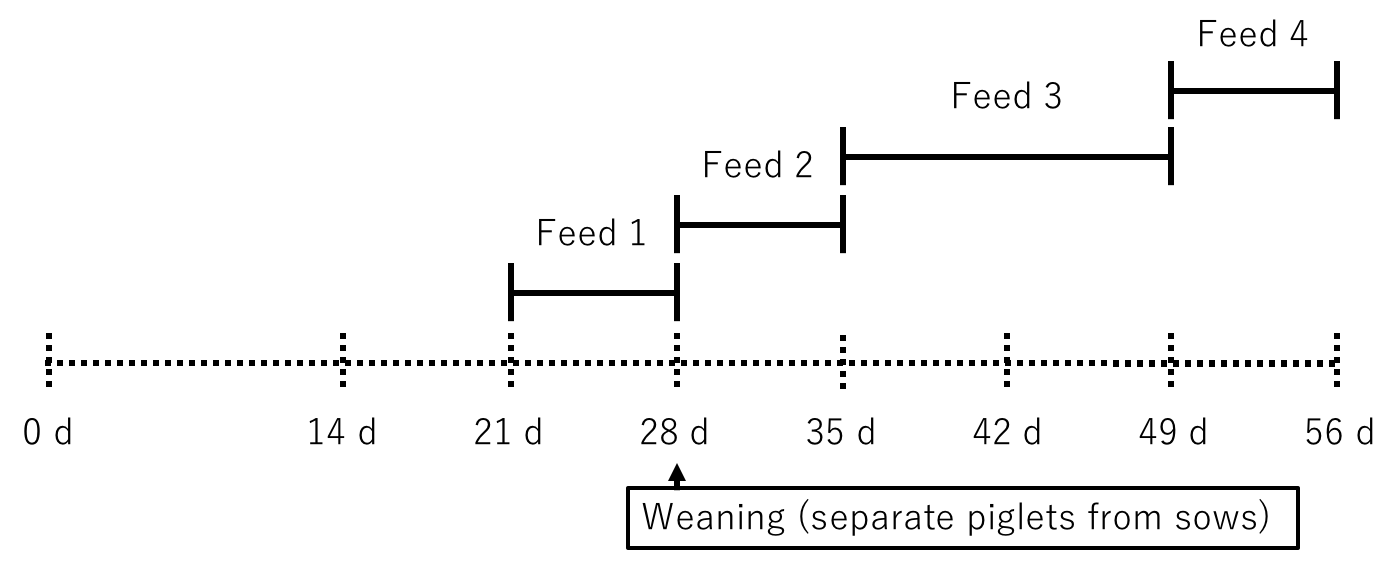


Figure S1. Feeding program of the experiment.

| Table S1. Composition of commercial feed. | | | | |
| --- | --- | --- | --- | --- |
| Chemical composition | Feed 1 | Feed 2 | Feed 3 | Feed 4 |
| Crude protein (%) | 24.5 | 21.0 | 20.0 | 18.5 |
| Crude fat (%) | 6.0 | 4.5 | 4.0 | 3.5 |
| Crude fiber (%) | 2.0 | 2.0 | 3.0 | 4.0 |
| Ash (%) | 7.0 | 8.0 | 7.0 | 6.5 |
| Digestible energy (Mcal/kg) | 4.0 | 3.8 | 3.7 | 3.5 |
| The components of the commercial feed was as follows:  Feed 1: animal origin feeds 49.0% (dried skim milk, whey protein concentrate, fish meal, dried whey, and plasma protein concentrate), grains 22.0% (bread crumbs, wheat feed flour, and potato starch), oil seed meals 3.0% (potato protein), others 26.0% (glucose, sugar, animal fat, lactose, and mineral and vitamin mix). Feed 2: animal origin feeds 36.0% (dried whey, dried skim milk, fish meal, whey protein concentrate, and plasma protein concentrate), grains 35.0% (corn, bread crumbs, wheat feed flour, dehulled soybean, and potato starch), oil seed meals 8.0% (potato protein, and soybean meal), brans 6.0% (rice bran separated with high temperature starch), others 15.0% (fructose, animal fat, glucose, sugar, and mineral and vitamin mix). Feed 3: grains 42.0% (corn, wheat, wheat feed flour, soybean, and potato starch), animal origin feeds 22.0% (dried whey, fish meal, and dried skim milk), oil seed meals 13.0% (soybean meal), brans 0.1% (rice bran), others 22.9% (bakery waste, yeast extracts, glucose, animal fat, and mineral and vitamin mix). Feed 4: grains 61.0% (corn, wheat, and dextrin), oil seed meals 22.0% (soybean meal), animal origin feeds 6.0% (fish meal, and dried skim milk), brans 0.1% (rice bran), others 10.9% (bakery waste, animal fat, lactose, and mineral and vitamin mix). | | | | |
